# Robotic Verticalization plus Music Therapy in Chronic Disorders of Consciousness: Promising Results from a Pilot Study

**DOI:** 10.3390/brainsci12081045

**Published:** 2022-08-06

**Authors:** Rosaria De Luca, Mirjam Bonanno, Giuliana Vermiglio, Giovanni Trombetta, Ersilia Andidero, Angelo Caminiti, Patrizia Pollicino, Carmela Rifici, Rocco Salvatore Calabrò

**Affiliations:** IRCCS Centro Neurolesi Bonino Pulejo, Via Palermo, SS 113, C. da Casazza, 98124 Messina, Italy

**Keywords:** minimally conscious state, robotic verticalization training, music stimulation, integrated strategic approach

## Abstract

Background: Music stimulation is considered a valuable form of intervention in disorders of consciousness (DoC); for instance, verticalization may improve motor and cognitive recovery. Our purpose is to investigate the effects of a novel rehabilitative approach combining robotic verticalization training (RVT) with personalized music stimulation in people with DoC. Methods: Sixteen subjects affected by minimally conscious state due to traumatic brain lesions who attended our Intensive Neuro-Rehabilitation Unit were enrolled in this randomized trial. They received either music robotic verticalization (MRV) using the Erigo device plus a personalized music playlist or only RVT without music stimuli. Each treatment was performed 2 times a week for 8 consecutive weeks in addition to standard neurorehabilitation. Results: We found significant improvements in all patients’ outcomes in the experimental group (who received MRV): Coma Recovery Scale-Revised (CRS-R) (*p* < 0.01), Level of Cognitive Functioning (LCF) (*p* < 0.02), Functional Independence Measure (FIM) (*p* < 0.03), Functional Communication Scale (FCS) (*p* < 0.007), Trunk Control Test (TCT) (*p* = 0.05). Significant differences between the two groups were also found in the main outcome measure CRS-R (*p* < 0.01) but not for TCT and FIM. Conclusions: Our study supports the safety and effectiveness of RVT with the Erigo device in chronic MCS, and the achievement of better outcomes when RVT is combined with music stimulation.

## 1. Introduction

Minimally conscious state (MCS) is a condition of severely altered consciousness in which a patient demonstrates inconsistent but definite, reproducible evidence of awareness of self or environment [1]. The evidence of awareness is explained by specific behaviors, including following simple commands, sustained visual fixation or tracking, reaching for or holding and manipulating objects, intelligible verbalization, verbal or gestural yes/no responses, and other behaviors that occur selectively in response to specific environmental events and cannot be accounted for by reflexive activity [2]. However, MCS must be distinguished from the vegetative state (VS), which has been replaced with the term unresponsive wakefulness syndrome (UWS). VS/UWS, unlike MCS, involves a lack of interaction with the surrounding environment; patients do not show self-awareness and cannot interact with other people [3].

Other specific cognitive and sensory-motor impairments of patients with chronic disorders of consciousness (DoC) are limb paralysis, loss of balance, agnosia, patient limited cooperation, deep sensory disorder, and/or ataxia as well as circulatory instability that may lead to a bedridden condition. Indeed, as DoC patients are more susceptible to severe medical complications, it is important to carry out early mobilization and frequent postural changes to avoid contractures and pressure ulcers and to stabilize vital signs (blood pressure, heart rate, and ventilation). Indeed, passive/active mobilization and verticalization (VT) procedures are the preliminary physiotherapy steps for the recovery of the patient [4,5]. In this clinical context, the process of gradual VT is introduced in the very early stage of the disease to avoid a deterioration of the autonomic nervous system as well as bedridden complications. VT activates proprioceptive, tactile, and vestibular pathways in comatose patients as well as in DoC, leading to an increased cortical area involved in trunk and lower limb control. Another mechanism that may contribute to the neurological improvement after head-up tilt could be the lowering of intracranial pressure [6]. Postural changes are known to alter intracranial pressure, modifying the venous outflow (and thus the cerebral blood volume) through the valveless jugular veins [7]. In fact, in order to avoid episodes of orthostatic hypotension, verticalization must be achieved with precocity and gradualness (from 0° to 45° and then up to 90°) and with a constant monitoring of blood pressure and heart rate [8]. It is demonstrated that robot-based neurorehabilitation improves motor performance by boosting brain plasticity, which plays a crucial role in motor and cognitive recovery. In particular, the Erigo device (Hocoma AG, Volketswil, Switzerland) combines progressive verticalization, cyclic leg movement (which allows for stepping reinforcement in combination with step synchronized muscle functional electrical stimulation, FES, at the lower limb), and body weight loading to ensure the safe stabilization of the patient in the upright position. Notwithstanding robotic VT (RVT), some authors [9,10] suggest the use of music presentation as well as an affective–emotional stimulus. In fact, listening to music is associated with a range of psychological and physical benefits, such as the reduction of pain, anxiety and agitation, in these patients. More specifically, music stimulation is thought to affect neural networks; accelerate brain plasticity; and increase the activity in frontal, temporal, parietal, and subcortical regions, with presumably important positive implications for the participants’ recovery process.

The aim of this study was to investigate the effects of an integrated rehabilitative approach combining robotic verticalization training (RVT) with music stimulation in people with DoC due to severe brain injury.

## 2. Materials and Methods

A total of 16 patients (11 males and 5 females) diagnosed with chronic MCS due to a traumatic etiology of brain damage and attending the Intensive NeuroRehabilitation Unit of the IRCCS Neurolesi Center Bonino Pulejo (Messina, Italy) from January 2021 to May 2022, were enrolled in this study. A more detailed description of the MCS patients’ demographic condition is reported in Table 1.

The IRCCS Neurolesi Center Bonino-Pulejo (Messina) carries out its activity focused on translational clinical research in the field of “Neuroscience” prevention, recovery, and treatment of severe acquired brain injury (SABI), including advanced rehabilitation using robotic systems and virtual reality. All experiments were conducted according to the ethical policies and procedures approved by the local ethics committee (IRCCS-ME 25/21). All patients’ legal guardians gave their written informed consent to study participation and data publication.

The inclusion criteria were: (i) age >18 years; (ii) diagnosis of chronic MCS (i.e., at least 6 months after the traumatic event) according to the Coma Recovery Scale Revised (CRS-R) [12,13], which is administered at enrollment; (iii) adequate pulmonary gas exchanging function (arterial O2 pressure/O2 flux ratio 250); (iv) stable hemodynamics (absence of dangerous variations of mean arterial pressure or heart rate), even if obtained with continuative amines support. The exclusion criteria were: (i) sedation; (ii) unstable intracranial pressure (ICP); (iii) cerebral perfusion pressure (CPP) <60 mmHg; (iv) fractures or skin lesions in thorax, abdomen, or lower limbs; (v) deep vein thrombosis; (vi) other medical conditions potentially interfering with verticalization; and vii) body weight >130 kg and height >210 cm.

Patients were randomly assigned to either the experimental group or the control group using a web-based application for block randomization (www.randomization.com (accessed on 15 March 2022). We used the block randomization method (block size = 4) in order to ensure balance in sample size across groups over time. The randomization procedure was run by a single investigator not involved in the clinical management of the patient. Only after the patient identifier number was communicated was the individual patient allocation revealed to the enrolling staff. The outcomes assessor was blinded to treatment allocation and to the study design, while the rest of the personnel involved in the study (physiotherapists, nurses, physicians) could not be.

### 2.1. Procedures

The enrolled patients received either RVT without music or with music stimulation (music robotic verticalization, MRV), both in addition to standard neurorehabilitation. Both treatments were provided in a dedicated room, 3 times a week, for about 8 consecutive weeks, each session lasting about 45 min. In addition to the experimental protocol, per standard care, each patient was followed by an interdisciplinary rehabilitation team, including neurologist, physiatrist, cognitive rehabilitation therapist, physiotherapist, speech therapist, rehabilitation nurse, and social worker, using an integrated specialist approach. Each participant was evaluated through the administration of specific clinical scales before (T0) and after each treatment (T1) by a blinded clinician.

### 2.2. Outcome Measures

We evaluated the patients’ cognitive and motor profiles in two separate phases, at the beginning and the end of either the RVT or the MRV. The motor-cognitive assessment included: (1) Levels of Cognitive Functioning (LCF) [14], one of the earlier developed scales for assessing cognitive functioning in post-coma patients; (2) the Coma Recovery Scale-Revised (CRS-R) [15], used to integrate neuropsychological and clinical assessment and validated for patients in VS/MCS as an appropriate measure for characterizing level of consciousness and for monitoring recovery of neurobehavioral functioning; (3) the Functional Independence Measure (FIM), an 18-item (13 motor [motFIM] and 5 cognitive [cognFIM]) measurement tool that explores an individual’s physical, psychological, and social function, used to determine the level of dependence of patients in daily life [16]; (4) the Trunk Control Test (TCT), used to evaluate motor impairment in a patient who has had a SABI [17]; and (5) the Functional Communication Scale (FCS) for the evaluation of communication abilities [18]. For more details, see Table 2.

### 2.3. Robotic Verticalization Training (RVT)

The Erigo enables a single therapist to provide mobilization, verticalization, and sensorimotor stimulation at the same time, safely and efficiently, combining gradual verticalization with robotic movement therapy [19,20]. Due to the unique afferent stimulation provided by the Erigo and the flexible harness, patients can be trained intensively and safely already in a very early stage of rehabilitation. The robotic leg movement and the cyclic leg loading offered by the device are critical afferent stimuli for the central nervous system. This leads to muscle activation and improved muscle pump function and venous return, ultimately resulting in improved cardiovascular stability [20]. Patients tolerate the upright position better than they do when treated on conventional tilt tables without a stepping function and cyclic leg loading. After the first session, aimed at adapting the patient to the device and lasting less than 25 min, each robotic session lasted about 45 min, during which the table inclination was gradually increased (from 45 to 90°) as well as the stepping velocity, according to the patient’s needs and clinical conditions. In the control intervention, patients received only RV training with the Erigo device without any sound stimulation, including noise or nonmusical sounds, in order to investigate only the effect of the Erigo itself.

### 2.4. Music Robotic Verticalization (MRV)

**Figure 1:**
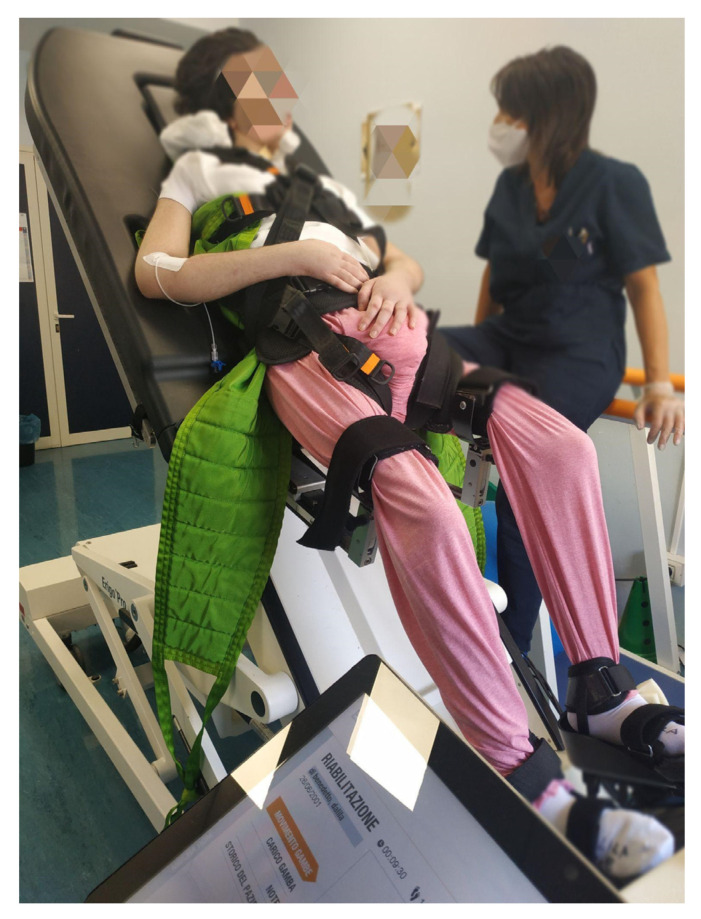
A patient with MCS during a music robotic verticalization session.

Patients with MCS in the EG received Erigo training sessions while listening to autobiographical/representative songs with headphones (see Figure 1), supervised by a therapist. During each RV session with the device, music was provided using a personal pen-drive connected to a musical player and containing fifteen songs, which were different in each patient according to the personal music (popular music, folk, rock, and classic music) he/she liked before the brain injury. These songs reminded the patients of significant emotional events. In detail, a psychiatric therapist administered a structured biographical interview to the caregiver of the patient with MCS (mother, father, daughter/son) in order to better understand his/her musical tastes. The main questions were about songs with the greatest emotional impact, were standardized by the rehab team, and included a part related to musical tastes and song titles; another part was about the patient’s general life experience. Data included work activities, events that have characterized the patient’s life, places of interest, personal identity (professional skills, habits, personal interests, values, eating habits, beliefs, leisure activities, etc.), the context of life (objects, environments, etc.), and relationships (friends, partners, other family figures, etc.) as well as traumatic and other significant life events (bereavement, marriage, etc.). This was extremely useful to the therapist in order to choose the most patient-tailored music therapy possible. Eventually, the intervention strategies relied on meaningful biographic associations among music, patients, and therapists.

### 2.5. Statistical Analysis

A sample size of convenience (8 + 8) was used as the study was regarded as a pilot trial with preliminary data. The statistical analysis of our data consists of a descriptive part in which we reported means and standard deviations (SD) for the numeric variables. The population studied is not normally distributed according to the result of the Shapiro–Wilk test. We also performed a first intra-group analysis at the beginning (T0) and at the end of the treatment (T1) using the Wilcoxon signed-rank test, a nonparametric test, assuming *p* value < 0.05. The analysis between the two groups (control and experimental) was performed using the two-tailed Mann–Whitney U test and assuming the *p*-value < 0.05. This test allowed us to find a difference between the mean of the ranks, and also to verify if there is evidence of a statistically significant difference between the medians of the two groups. All analyses were performed on software R, version 4.1.3 [21].

## 3. Results

All of the MCS patients completed the study protocol without any side effects. In particular, there was no need to call either nurses or physicians to manage hypotension or other medical problems that could have potentially occurred during the training. Comparing the clinical and psychometric test scores between baseline and follow-up, we found significant changes in all patients’ outcomes, especially in the experimental group: CRS-R (*p* < 0.01), LCF (*p* = 0.02), FIM (*p* = 0.03), FCS (*p* < 0.007), TCT (*p* = 0.05). These data showed improvements in awareness, global cognitive functioning, functional status, nonverbal communication skills, and trunk control.

On the other hand, in the control group, although the individual scores of the outcome measures improved with standard RV, the improvements did not reach statistical significance. Furthermore, we noted statistically significant differences between the EG and CG in all outcome measures except for TCT and FIM. The improvements to be highlighted are especially in the CRS-R (*p* < 0.01), LCF (*p* = 0.03), and FCS (*p* < 0.01) scores as shown in Table 3.

## 4. Discussion

One of the most emotional and salient stimuli of our environment is probably music, and this is why in our study, songs were accurately selected based on the patient’s preference and emotional involvement. The effects of music interventions including active and receptive music therapy for people living with DOC have been subject to empirical studies in the past. The therapeutic effects of music are being recognized increasingly in the field of rehabilitation medicine. Moreover, patients with SABI need to achieve early verticalization and improve arousal. Indeed, robotic devices have been successfully applied in motor training during neurorehabilitation to improve motor function and motivation. The collaboration between music and robotic training in neurorehabilitation has not been investigated with clinical trials, and the literature evidence is poor. Recently, Vinolo-Gil et al. (2021) showed that the combination of music therapy and physiotherapy, as a different therapeutic strategy, can be effective in subjects with cerebral palsy to improve motor functions [22]. Baur et al. investigated the combination of music and activities promoting creativity in motor training, and thus intrinsic motivation, in subjects with neuromuscular disease performing robot-assisted training for the arm with the upper limb robotic exoskeleton ARMin [23]. Recently, several studies have shown that music can boost cognitive functions in both normal and brain-damaged subjects. However, few studies have suggested a beneficial effect of music in patients with a DoC, and it is difficult to reach a conclusion since they did not use constantly quantified measures and a control condition/group [24,25]. Our study supports the safety and the effectiveness of ERIGO verticalization in bedridden patients with DoC and the achievement of better outcomes when VRT is combined with music stimulation. Indeed, both groups completed the training without side effects and improved after robotic verticalization, although the control group did not achieve statistically significant differences. To the best of our knowledge, this is the first study that investigates the role of music to improve awareness (per CRS-R) and global nonverbal communication skills (per FCS) as well as motor scores (per TCT). However, our data, especially for the CRS-R, have to be interpreted cautiously due to heterogeneity of the sample at the baseline. Previous clinical experiences have shown that the unique afferent input provided by the Erigo can have a positive effect on the patient’s consciousness, body awareness, and intestinal activity in neurological diseases [26]. Calabro’ et al. found that the Erigo device could be a safe rehabilitative strategy for improving motor and cognitive functions and thus avoiding a chronic bedridden condition owing to its potential for improving functional plasticity within sensory–motor and vestibular systems [27]. In our innovative approach, during the training with the device, we applied music as an autobiographical stimulus that was able to better involve MCS patients in the rehabilitative activities, since listening to emotional music may create connections with a patient’s background and life history, increasing motivation and participation. In fact, this was possible, in our opinion, only because the music reminded the participants of specific emotional events and that neutral songs would have not led to the same improvement.

Our promising results confirmed current literature findings indicating that music might play a critical role on the functional scale, general physical indexes, and favorable outcomes for DOC patients [28,29,30]. The self-referential and autobiographical properties of music are able to boost perception and cognition in patients with DOC and could also serve as a prognostic tool [31,32]. Indeed, a recent study by Martinez-Molina et al. demonstrates that music therapy improves executive function in patients with traumatic brain injury; moreover, their data show that morphometric and resting-state functional connectivity are sensitive markers for monitoring the neuroplasticity (especially within the right inferior frontal gyrus) induced by music training [33]. Some studies carried out in DOC populations showed that music enhanced arousal and attention when compared with white noise and disliked music [34]. We preferred not to use any kind of noises/music in the control group as we wanted to investigate the real effect of the Erigo alone, avoiding other additional/misleading stimuli.

Other evidence also suggests a potential impact of music therapy on consciousness recovery [35], as confirmed by our results. We supposed that the positive effects of music on cognitive functions of patients with DOC might be explained by an overall cortical arousal and/or an awareness enhancement. This is in agreement with the arousal and mood hypothesis, suggesting that the effect of music listening on cognitive abilities can be attributed to changes in listeners’ arousal and mood [36]. The beneficial effects of music might be also associated with its engagement properties. Indeed, two types of brain networks interact when listening to music, the external frontoparietal network and the internal network (precuneus/posterior cingulate, mesas-frontal/anterior cingulate, and temporo-parietal cortices), have been shown to have a correlation that decreases with an increasing level of consciousness [37]. Thus, it could be suggested that the effect of music on cerebral processes in DOC patients might reflect the music’s capacity to act on these two major networks to support consciousness. These promising findings should now be extended with an increased number of patients to validate the hypothesis of the beneficial effect of music during RVT on cognition as well as sensory–motor outcomes in patients with DoC.

Our study has some limitations to acknowledge. First, the small sample size prevents us from generalizing the results to the larger DoC population. Second, we did not investigate long-term aftereffects, so we are not able to state if and to what extent the improvement in cognitive function lasts. Third, the randomization of the study population was not carried out in relation to education level but instead in the simple order of each patient’s enrollment; thus, it was by pure chance that the experimental group contained a more educated population than the control one. In fact, education could be a positive prognostic factor in recovery and may have biased the results. Additionally, future studies could focus on the effects of neutral music stimuli compared with the significant emotional stimulation of patient-tailored music and the impacts of these different stimuli (emotional and not) on the global MCS patient’s neurorehabilitation and recovery. Furthermore, our analysis of the baseline data showed no statistically significant differences between the two groups except for the CRS-R scores, which were higher in the EG than the CG, and this may have somehow affected the results. This should be confirmed using a more homogeneous sample with a parallelization approach. Finally, no objective measure of functional recovery, including neuroimaging or functional connectivity, was investigated.

Nonetheless, this should be considered a pilot exploratory study that might break ground on further research investigating the promising effects of using coupled approaches with DoC patients. Further studies with larger samples, higher-quality methodology, and long-term follow-up are needed to confirm the therapeutic effects of this new combined approach.

## 5. Conclusions

In conclusion, this pilot study suggests that patients with Doc may benefit from innovative training and that robotic verticalization plus music may potentiate cognitive function in these very frail and vulnerable individuals.

## Figures and Tables

**Table 1:** Demographic and Clinical Description.

**Control Group (CG)**	**All**	**Males**	**MCS Level**	**Age**	**Females**	**MCS** **Level**	**Age**
Patients	8	6 (75.00)			2 (15.00)		
CRS-R baseline	8 ± 3.11	6	-	75	6	-	64
		9	+	69	9	+	55
		7	-	65			
		15	+	54			
		6	-	73			
		6	-	73			
Time elapsed since injury (months)	9.12 ± 2.16	10			9		
		6			12		
		7					
		8					
		12					
		9					
Age (years)	66 (±8.08)	69.83 (±4.57)			54.5 (±0.70)		
Education Elementary school Middle school High school University	2 (25) 5 (62.5) 1 (12.5) 0 (0.00)	1 (16.66) 5 (83.34) 0 (0.00) 0 (0.00)			1 (50.00) 0 (0.00) 1 (50.00) 0 (0.00)		
**Experimental Group (EG)**	**All**	**Males**	**MCS Level**	**Age**	**Females**	**MCS**	**Age**
Patients	8	5 (62.5)			3 (37.5)		
CRS-R baseline	10.6 ± 2.32	12	+	57	10	+	73
		8	-	35	10	+	45
		12	+	62	10	+	65
		8	-	58			
		15	+	60			
Time elapsed since injury	9.25 ± 2.05	9			6		
		9			12		
		8			8		
		12					
		10					
Age (years)	56.87 (±11.84)	54.4 (±11.01)			61 (±14.42)		
Education							
Elementary school	2 (25.00)	1 (20.00)			1 (33.3)		
Middle school	3 (37.5)	2 (40.00)			1 (33.3)		
High school	2 (25.00)	1 (20.00)			1 (33.3)		
University	1 (12.25)	1 (20.00)			0 (0.00)		

Legend: CRS-R scores and continuous variables are expressed in mean and standard deviation (±), whereas categorical variables as frequencies and percentages. + indicates MCS Plus (patients with specific pivotal behaviors, such as consistent and reproducible movement to command, object recognition and intelligible verbalization with intentional (nonfunctional) communication) [11]; - indicates MCS Minus (patients with reaching, visual pursuit, fixation, object manipulation, and automatic motor response).

**Table 2:** Motor-cognitive assessments for patients with MCS.

Test/Scale	Domains	Description
Levels of Cognitive Functioning (LCF)	Cognitive Functioning	Each level of LCF is presented as a behavioral description in 10 narrative forms, and the physician must decide which level best describes the patient’s present behaviors: Level I: No response–Total assistance; Level II: Generalized Response–Total Assistance; Level III: Localized Response–Total Assistance; Level IV: Confused/Agitated–Maximal Assistance; Level V: Confused, Inappropriate Non-Agitated–Maximal Assistance; Level VI: Confused, Appropriate–Moderate Assistance Level VII: Automatic, Appropriate–Minimal Assistance for Daily Living Skills; Level VIII: Purposeful, Appropriate–Stand By Assistance; Level IX: Purposeful, Appropriate–Stand By Assistance on Request; Level X: Purposeful, Appropriate–Modified Independent;
Coma Recovery Scale -Revised (CRS-R)	Consciousness State	The Italian version of the Coma Recovery Scale-Revised (CRS-R), a reliable tool that can distinguish patients in the minimally conscious state from those in a vegetative state. The CRS-R consists of 29 hierarchically organized items divided into 6 subscales addressing auditory, visual, motor, oromotor, communication, and arousal processes. It is designed to detect subtle changes in neurobehavioral status and to predict outcomes in patients with severe alterations of consciousness.
Functional Communication Scale (FCS)	Communicative Functioning	The FCS is a specialist language questionnaire of verbal and nonverbal abilities for investigating global communication by evaluating language abilities (verbal and nonverbal communication skills). It is carried out by the speech therapist to investigate motivation, collaboration, understanding, and language abilities. Response options range from 0 to 22.
Functional Independence Measure (FIM)	Functional Status	The FIM is an ordinal scale composed of 18 items with 7 levels ranging from 1 (total dependence) to 7 (total independence) designed to determine the level of disability of patients, as reflected in their need for assistance and/or aids during the execution of activities of daily living. The FIM can be subdivided into a 13-item motor subscale (motFIM) and a 5-item cognitive subscale (cognFIM). The ranges of scoring for the motor and cognitive subscales are 13 to 91 and 5 to 35, respectively. gGood interrater reliability has been demonstrated both for the TCT and for the FIM.
Trunk Control Test (TCT)	Trunk Movement Patterns	The TCT examines four axial movements: rolling from a supine position to the weak side (T1) and to the strong side (T2), sitting up from a lying-down position (T3), and sitting in a balanced position on the edge of the bed with feet off the ground for 30 s (T4). The scoring is as follows: 0, unable to perform movement without assistance; 12, able to perform movement but in an abnormal manner; and 25, able to complete movement normally. The TCT score is the sum of the scores obtained on the four tests (range, 0 to 100). The examiner’s score must relate solely to the performance during the test and not be based on referred data.

**Table 3:** Statistical comparison of the clinical score variations from baseline to follow-up between experimental group (music robotic verticalization) and control group (robotic verticalization); scores are in median (first–third quartiles).

Clinical and Psychometric Scales	Music Robotic Verticalization (Median, First-Third Quartile)	Robotic Verticalization (Median, First-Third Quartile)	*p*-Value
CRS-R			
T0	10 (9.5–12)	6.5 (6–9)	0.03
T1	14.5 (13.5–15.25)	7.5(6–9.25)	0.01
LCF			
T0	4.5 (3.75–5)	1.5 (1–2.75)	0.052
T1	7 (6–7.25)	2 (1.75–3.25)	0.03
FIM			
T0	21.5 (18–24.5)	18 (18–19)	0.24
T1	25 (21.75–46.25)	18 (18–18.75)	0.06
TCT			
T0	12 (0–18)	0 (0–0)	0.14
T1	14.5 (0–30)	0 (0–11.25)	0.29
FCS			
T0	24 (20.75–26.5)	17.5(16.75–23.25)	0.14
T1	30.5 (23.75–44.5)	17.5 (16.5–21.25)	0.01

Legend: CRS-R (Coma Recovery Scale–Revised); LCF (Levels of Cognitive Functioning); FIM (Functional Independence Measure); Trunk Control Test (TCT); FCS (Functional Communication Scale).

## Data Availability

Data will be available on request to the corresponding author.
